# Addressing false information through local capacity building in community-based disaster risk management

**DOI:** 10.4102/jamba.v17i1.1836

**Published:** 2025-04-14

**Authors:** Jefferson M. Cuadra, Vincent N. Cotoron

**Affiliations:** 1 Department of General Education, Faculty of Social Sciences, Caraga State University Cabadbaran Campus, Cabadbaran, Philippines; 2 Department of Environmental Sciences, Faculty of Forestry and Environmental Sciences, Caraga State University, Butuan, Philippines

**Keywords:** local capacity building, false information, disaster risk management, digital literacy, disaster response

## Abstract

The increase in climate-related disasters makes it necessary to address the spread of false information for effective disaster management. While social media can speed up disaster information, false information causes anxiety, fear, and poor decision-making, which affect preparedness and resilience. This study describes how local capacity building can address false information in community-based disaster risk management. The research uses a qualitative approach with secondary data analysis and an integrative literature review. The findings of this study show that there is a need to strengthen local capacity building to prevent the spread of false disaster information to communities and interested parties. The current response to misinformation in disaster situations includes digital literacy and media literacy education to help individuals assess news sources and verify information. It also includes community-based training programmes focusing on disaster preparedness, response, and communication, aiming to reduce the impact of misinformation. Establishing local communication networks, such as community radio stations or messaging groups for reliable alternatives to social media, is also critical to address misinformation. The study also found that trusted local leaders and influencers ensure accuracy, and transparent official channels promote public trust. Furthermore, simulated drills and workshops teach information verification practices, preparing communities to manage misinformation during crises. These efforts, along with local capacity building, are essential in strengthening community resilience and improving disaster management practices.

**Contribution:**

This study contributed to improving disaster management communication with local communities. It strengthened the coordination of disaster information and improved partnerships among stakeholders. It also enhanced collaboration and addressed communication gaps in disaster response.

## Introduction

As climate change makes natural hazards more frequent and severe, it is integral for communities to focus on effective disaster risk management (DRM) strategies that blend traditional practices with modern tools (Scolobig et al. 2015). In this era of technology and social media, while these platforms submit valuable information, they also pose a risk by spreading false information – called hoaxes – that can sabotage disaster preparedness and response (Eichensehr & Citron 2024). Disinformation is false or misleading content disseminated to deceive and cause public harm (Zhang & Ghorbani 2020). As opposed to misinformation, which refers to false or misleading information not intended to intentionally deceive, manipulate or harm a person, social group, organisation or country (Molina et al. 2021). It does not create or falsify the initial content. This division of the type of false content is part of the Organization for Economic Cooperation and Development (OECD) typology of online untruth, including a range of digital content that is false and misleading (De Cock Buning 2018).

**FIGURE 1:**
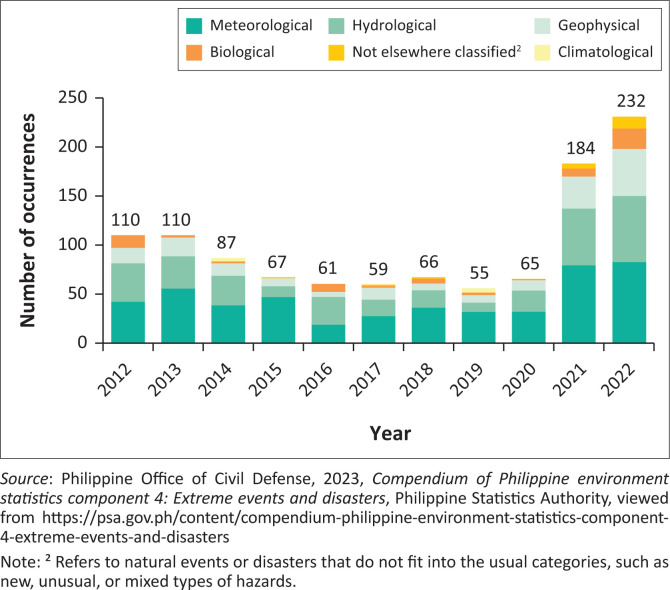
Documented cases of natural extreme events and disasters, 2012–2022.

In a disaster-prone country such as the Philippines, disaster preparedness is also determined by receiving information as a source of knowledge and as a basis for anticipatory decisions that must be made. Based on the data from the Philippine Statistics Office from 2012 to 2022 and the beginning of the coronavirus disease 2019 (COVID-19) pandemic (non-natural disasters), Figure 1 shows the type of natural extreme events and disasters that occurred from 2012 to 2022. It illustrates the number of occurrences of various types of events from 2012 to 2022, categorised into meteorological, hydrological, geophysical, biological, climatological and others. In 2012 and 2013, there were 110 occurrences each, dominated by meteorological and hydrological events. From 2014 to 2019, the number of events gradually decreased, reaching a low of 55 in 2019. However, in 2020, the occurrences rose slightly to 65, followed by a significant spike in 2021 and 2022, with 184 and 232 occurrences, respectively. This sharp increase in the later years is particularly evident in the meteorological and climatological categories, indicating a rising trend in these types of events (Philippine Office of Civil Defense 2023).

As shown in Figure 1, the types of natural extreme events and disasters that occurred in the Philippines were hydro-meteorological disasters (floods, droughts, tropical cyclones, storm surges) and geophysical disasters (earthquakes, tsunamis and landslides) related to climate change and global warming. The disasters had a broad impact of damage and danger to local communities. The impact includes destruction of homes, critical infrastructure and death (Schuette 2019).

Even before the pandemic, Filipinos had already recognised the importance of information and communication technologies (ICTs) such as personal computers, mobile phones, the Internet, emails and social media platforms (Albert et al. 2021). With 76 million active users – accounting for 71% of the population according to The Asean Post – these tools became even more critical during the COVID-19 pandemic. However, the surge in digital technology and social media use also marks a darker side: the spread of misinformation, which fuelled anxiety and fear amid the uncertainty of the crisis. December 2021 Social Weather Stations survey revealed that 69% of adult Filipinos consider fake news in the media to be a serious problem. In addition, over half (51%) of Filipinos reported finding it difficult to identify fake news on television, radio or social media (Dang 2021).

Efforts to prevent the spread of false information about disasters on social media are important for stakeholders, particulalrly within communities (Yu et al. 2018; Roopnarine et al. 2021) the increasing frequency of disasters driven by climate change, ensuring accurate information is essential for effective disaster management (Muhame, Ncube & Bahta 2024). While social media can be a powerful tool for quickly disseminating information during emergencies, the spread of misinformation can lead to heightened anxiety, fear, poor decision-making, subversion community preparedness and resilience (Skiba 2024). From a sociological perspective, the way communities respond to disaster information is deeply influenced by trust in sources and the collective ability to discern credible information. Misinformation can erode social trust and cohesion, making it harder for communities to act collectively in times of crisis (Shahbazi & Bunker 2024).

This study explores how connecting different aspects of information can enhance knowledge and preparedness in DRM. The DRM involves a combination of prevention, mitigation and preparedness efforts, along with emergency responses before, during and after disasters. It includes two main components: disaster management and disaster risk reduction. Disaster management covers the planning and coordination needed at every stage of a disaster – before it happens, while it is occurring and in its aftermath. Disaster risk reduction focusses on minimising hazards and potential risks but does not address the management of disasters or their impacts.

**FIGURE 2:**
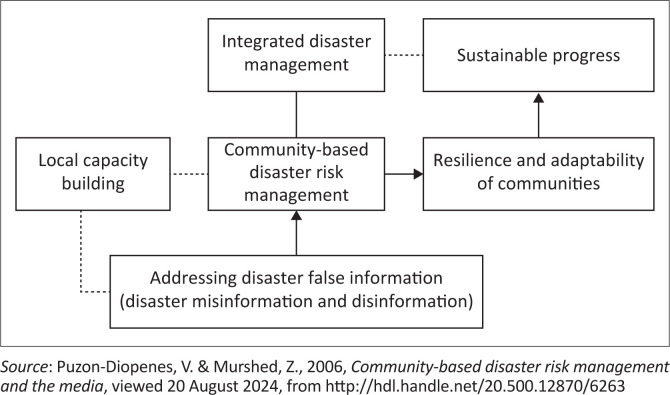
Conceptual framework of the study.

Figure 2 presents a conceptual framework that relates to the challenges discussed in this study, particularly the role of managing false information during disasters. It emphasises the need for effective information management as part of a comprehensive approach to disaster management.

Building community resilience and adaptability to disasters involves integrating key elements of capacity building, such as effectively managing and distributing accurate information about disasters. In these situations, applying situational crisis communication theory can help address the risks associated with rumours and misinformation. This approach involves actively rejecting false information, refuting rumours and challenging their sources, which can help counteract the spread of misleading information. Social capital theory emphasises the importance of networks, relationships and trust within communities to facilitate collective action and support during crises. Collective efficacy theory highlights the community’s shared belief in its ability to act together effectively to address challenges and achieve common goals.

Incorporating local capacity-building efforts into community-based DRM can reduce vulnerability and manage uncertainty more effectively. Integrating strategies to address false information about disasters into DRM enhances community resilience and adaptation, thereby supporting sustainable progress.

## Research methods and design

This study employs a qualitative approach to analyse secondary data and conduct an integrative literature review. An integrative review summarises past empirical or theoretical literature to provide a comprehensive understanding of a specific healthcare issue (Hopia, Latvala & Liimatainen 2016). Integrative reviews help build nursing science by informing research, practice and policy. This review method allows for the inclusion of both experimental and non-experimental research (Whittemore et al. 2005; Whittemore & Knafl 2005). Secondary data were gathered through document studies, specifically examining disaster-related misinformation and disinformation debunked by Rappler in 2023 and reports from the Philippine Atmospheric Geophysical and Astronomical Services Administration (PAGASA) and DOST-PHIVOLCS. The analysis centres on the spread of false information about disasters that occurred in 2023 and examines them. The decision to focus on data from 2023 was made to map the types of disasters and the role of social media in spreading misinformation pre- and post-COVID-19 pandemic.

**FIGURE 3:**
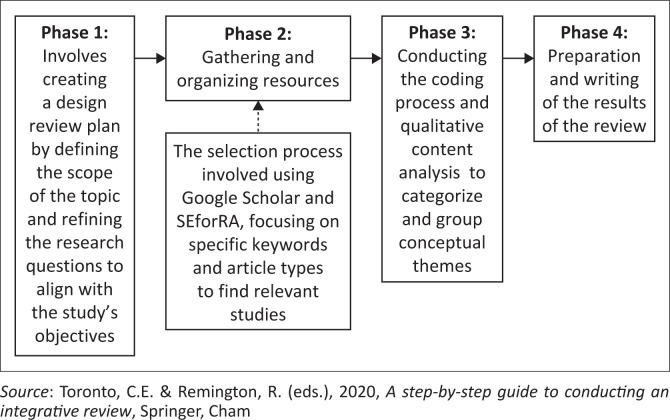
Review process.

The integrative literature review involved selecting and incorporating perspectives from various fields related to misinformation and community-based DRM. The goal was to reconceptualise and integrate these concepts effectively. As shown in Figure 3, documents for the review were sourced online using Google Scholar and SEforRA (Search Engine for Research Articles). Keywords such as ‘natural hazard, disaster false information’, ‘misinformation’, ‘disaster management’ and ‘risk reduction’ and sociology of disasters’ guided the search. The selection process focussed on qualitative journal articles published between 2019 and 2023. From this, 26 relevant documents were chosen for review. The initial search resulted in a sample of 429 articles. In light of the inclusion and exclusion criteria, 17 studies remained in our review. These documents were then analysed through a coding process, allowing for an exploration of the interrelationships between concepts within the study’s conceptual framework and objectives.

### Measures

In the current review, we assessed the quality of studies using the American Association of Critical-care Nursing (AACN) revised evidence-levelling system, which sorts evidence into six levels. At the top, Level A includes the strongest evidence from meta-analyses and meta-syntheses. Level B features well-designed controlled studies, both randomised and non-randomised. Level C covers a mix of qualitative studies, descriptive or correlational studies, integrative reviews, systematic reviews and randomised controlled trials with inconsistent results. Level D is reserved for peer-reviewed professional standards. Level E represents theory-based evidence from multiple-case reports and expert opinions. Finally, Level M is used for recommendations from manufacturers (Armola et al. 2009).

### Ethical considerations

This article does not contain any studies involving human participants performed by any of the authors.

## Results

The findings reveal that managing false information is essential for developing trustworthy knowledge and strengthening disaster adaptation capabilities, which are both critical aspects for DRM. Climate change is intensifying disaster risks by increasing the frequency of weather and climate-related hazards while also making communities more vulnerable through ecosystem degradation and decreased access to water and food. As illustrated in Table 1, false information is spread through social media platforms, misrepresenting the type, impact and location of disasters. This misinformation can amplify fear, particularly among those already traumatised or affected by previous disasters, thereby heightening risks for vulnerable groups.

**TABLE 1:** Analysis results of false information content regarding natural disasters in 2023.

Clarification of false information (fact checking)	Content of false information			Media used for dissemination of content	Actual conditions
	Disaster location	Disaster type	Disaster impact		
March 2023	Some parts of Davao and nearby cities	Earthquake because of volcanic eruption	Warning of a bigger quake with an epicentre	Facebook	There was no catastrophic event
June 2023	Metro Manila	Earthquake	Imminent quake to hit	Facebook	There was no catastrophic event
June 2023	Catbalogan City, Samar	Typhoon	Strong typhoon same as Typhoon Yolanda	Facebook	The LPA has a slim chance of developing into a tropical cyclone, but there was no catastrophic event that happened
July 2023	Bacolod	Earthquake	Preparing for a Magnitude 8 Earthquake	Facebook	There was no catastrophic event
November 2023	Cagayan de Oro City and Tagoloan	Earthquake	Quakes jolted in Mindanao	Facebook	There was no catastrophic event
July 2023	Northern Luzon	Earthquake	An 8.1 magnitude earthquake was expected	Facebook	There is a 7.0 magnitude earthquake, but the PHILVOLCS claimed it as aftershocks, and no catastrophic event happened after
July 2023	Entire Philippines	Storm, typhoon, floods	New Strong storm and called ‘Duterte’	Facebook	There was no catastrophic event
November 2023	Bohol	Earthquake	Two earthquakes will hit Bohol	Facebook	There was no catastrophic event
December 2023	Surigao City	Tsunami and earthquake	Surigao City hit tsunami and following a 7.2 magnitude earthquake	Facebook	There was no catastrophic event

*Source:* Pasion, L., 2023, ‘Secondary data analysis’, *Disaster-related lies, disinformation debunked by Rappler in 2023*, viewed 20 August 2024, from https://www.rappler.com/environment/disasters/lies-disinformation-related-disasters-debunked-2023/

### Analytic strategy

The data were analysed with consideration of purpose, methods and findings of the reviewed studies. Considering the main findings, this examines the role of managing false information during disasters and how it was identified and summarised. Then, based on common meanings and central issues of these findings, they were organised and integrated as categories and themes. A summary of one main category and their themes that emerged are presented in Table 2.

**TABLE 2:** Summary.

Category	Theme	Findings
Managing false information in disaster response	Impact of false information on disaster management	Confusion, rumours and panic Increased vulnerabilityHindered response efficiency Erosion of trust
	Strategies for managing and correcting false information	Information Verification Mechanisms Education and awareness campaigns Integration with existing DRM systems

DRM, disaster risk management.

## Discussion

Managing false information is part of building reliable and trustworthy knowledge for disaster adaptation capabilities and risk management. As alluded by Scolobig et al. (2015), climate change is raising disaster risk through increased weather hazards and greater vulnerability of communities because of ecosystem degradation and reduced water and food availability. As shown in Table 1, false information spreads inaccuracies about disaster types, impacts and locations across various social media platforms. This study concurs with Molina et al. (2021) that misinformation increases fear, especially for those previously traumatised by disasters, and poses risks to vulnerable groups. Furthermore, the spread of false information during disasters creates confusion and panic, disrupting coordinated responses (Setten & Lein 2019; Oktari et al. 2023). Early sociological studies, such as Barlow and Craske (2013) describe panic as an escape response to immediate danger under unpredictable conditions. The reliability of social media as a source during emergencies is often questioned. In crises, communities lack localised, actionable information, leading to information gaps easily filled by misinformation or ‘improvised news’ (Arif et al. 2016). Misinformation spread through social media can weaken and delay rescue operations (Muhammed & Mathew 2022). In addition, community members in affected areas, acting as first-hand reporters, can unintentionally harm rescue efforts by spreading unverified information (Okwori 2020).

Managing misinformation during disasters requires strengthening the credibility of official channels, increasing media literacy and implementing strategies to counter misinformation in real time (Dallo et al. 2023). Gottschalk (2022) describes rumouring as a collective exchange to understand crisis situations. Social media, prone to both scarcity and overload of information, can amplify misinformation. Anxiety, information source uncertainty, personal involvement and social ties contribute to rumor spreading during crises (Blaustein et al. 2023). Anxiety, defined as a negative emotional state from distress, drives rumors when reliable information is lacking or overwhelming (Imperiale & Vanclay 2019). In such cases, people may turn to rumours as their main information source. Zhang and Ghorbani (2020) found that users are more likely to share misinformation they perceive as credible, especially if it appears threatening. However, repetition from multiple sources increases its believability although recognising the misinformation reduces the likelihood of further sharing (Pennycook, Cannon & Rand 2018).

Recent studies have increasingly emphasised the role of social systems in creating risks, shifting focus from disaster agents to vulnerabilities. Singh, Eghdami and Singh (2014) argue that societal dimensions are integral to hazard analysis, underlining how social structures contribute to disaster vulnerability. Similarly, Aksha et al. (2019) developed a Social Vulnerability Index to assess how numerous social factors, such as socioeconomic status and housing characteristics, influence communities’ susceptibility to environmental hazards. Lillywhite and Wolbring (2022) examined the vulnerabilities of children with disabilities in disaster contexts, emphasising the need for inclusive emergency planning that accounts for social disparities. These studies collectively underline the importance of understanding and addressing the social dimensions of vulnerability to enhance disaster resilience. Alexander (2018) defines disaster as an event disrupting social systems, requiring interventions to restore order. It links disasters to the interaction between hazardous events and vulnerable populations. Albertson Fineman (2017) argues that vulnerability stems from weaknesses in social structures, not from the events themselves. Vulnerability relates to poverty but also arises from social factor such as isolation, insecurity and limited access to resources (Islam & Walkerden 2022; Rucinska 2019). Risk exposure varies with social identity, gender, age and other factors (Hagley 2021; Usta et al. 2024; Turan & Oral 2023; Petraroli & Baars 2022).

Managing false information during disaster response increases these vulnerabilities (Marchezini et al. 2025; Xie & Chen 2024). Misinformation causes confusion, disrupts communication and leads to poor decisions, slowing response efforts (Lewandowsky, Ecker & Cook 2017). Vulnerable groups, especially those with limited access to verified information, face greater risks. False information reduces trust in official sources, hindering coordination between responders and communities (McEntire 2021).

Local capacity building in DRM allows communities to confirm circulating false information more effectively. Using the local language also helps clarify disaster risks and manage crises (Fresnoza 2021). However, knowledge gap exists in how communities use their information networks to support formal disaster management processes. Emergency response agencies must improve methods to capture and use information from these community networks for disaster management and risk reduction that better fit local needs (Domingo & Manejar 2018; Torpan et al. 2021; Wang et al. 2019).

Community-based DRM involves affected communities directly in hazard assessments, vulnerability and capacity evaluations and the planning, implementing, monitoring and evaluating of local disaster risk reduction efforts. These actions align with Sustainable Development Goal 13, which calls for urgent steps to address climate change and its impacts. Disaster risk reduction aims to lessen hazard impacts and address social, economic and environmental conditions that contribute to these risks. Disaster resilience and coping skills are strengthened through timely information and connections during emergencies. False information disrupts community disaster preparedness and adaptation efforts, leading to bias, misinformation and psychological health issues (Awuh, Mallick & Mairomi 2022). Managing information and knowledge through social media and digital tools requires careful measures to prevent the misuse of technology in disaster management contexts.

Regulating disaster information on social networking sites allows these platforms to support every stage of the DRM cycle (Reuter, Hughes & Kaufhold 2018). During the mitigation phase, social media educates the public on various risks and encourages knowledge sharing for disaster adaptation. In the preparedness phase, social media broadcasts warnings and updates increasing situational awareness. In the response phase, social media shares real-time information, helps locate victims and relatives and builds disaster maps, enabling users to coordinate aid efforts and provide eyewitness information. During the recovery stage, social media aids community organisation by sharing relevant resources and support information (Zobeidi et al. 2024).

Local capacity building support coordination through establishing local leadership, mobilising communities with local media and enhancing communication within communities (Oktari 2023; Wang et al. 2019). Supporting local initiatives helps combat disinformation by using human-centered digital approach that addresses the needs of each communities (Shakeri et al. 2021; Rumpa et al. 2023). Efforts to mitigate misinformation on social media focus on establishing initial communication from authorised officials and using scientific evidence. Effective strategies to limit misinformation include coordinating responses, verifying sender and source credibility, directly communicating with vulnerable groups, providing digital literacy education and clarifying uncertainties based on scientific data. Building local capacity to select credible information and prevent false information is critical. Digital literacy education is a priority in the preparation and mitigation phases for communities managing disaster risk.

The Sendai Framework calls for collaboration among government and stakeholders to identify disasters, manage risks and build community resilience. Empowering local communities requires coordinated efforts to counter false information and improve disaster communication, involving key actors such as disaster survivors, local media managers, community leaders, Non-Government Organisation (NGOs), business owners and local authorities. The framework outlines three priorities: strengthening disaster risk governance, investing in risk reduction to build resilience and enhancing preparedness for effective response and recovery (Surianto et al. 2019). This approach aims to support communities in ‘Building Back Better’ during recovery, rehabilitation and reconstruction. The academic sector and government collaborate to strengthen disaster resilience by identifying risks, educating, managing information and empowering communities. The government can implement the Sendai Framework at the local level by aligning it with sustainable development goals. This alignment, along with engaging communities in information and knowledge management, supports disaster risk reduction and prepares communities for disaster risks. This approach builds local capacity for effective disaster anticipation and adaptation (Parsons et al. 2016).

## Conclusion

This study identifies the need to integrate local capacity building into DRM to counter false information. Effective information management during emergencies requires digital literacy and disaster communication education tailored to local conditions. Community-based DRM should focus on community perspectives and involve multi-stakeholder collaboration to reach all vulnerable groups. The research presents a holistic approach to disaster management and communication, encouraging coordination and partnerships among stakeholders. It highlights local capacity building through information management strategy for sustainable development, addressing economic, social-cultural and environmental factors before, during and after disasters. The study recommends evaluating DRM practices and updating policies to enhance local community participation in disaster adaptation.

Beyond digital literacy, other strategies for combating false information in disaster management include community-based training in disaster preparedness, response and communication. Media literacy education helps individuals assess news sources and identify reliable information. Local communication networks, such as community radio or messaging groups, provide alternatives to social media and share verified updates. Trusted local leaders and influencers can reduce reliance on unverified content. Promoting transparency in official channels ensures timely and clear updates from disaster management agencies, fostering public trust. Simulated drills and workshops teach information verification, preparing individuals to reject false claims during crises. Developing community resilience frameworks ensures a collective understanding of disaster response, reducing confusion and misinformation. These strategies, alongside digital literacy, strengthen communities’ ability to manage misinformation risks during disasters.
